# 

**Published:** 1889-03

**Authors:** 


					﻿TWENTY EXTRA PACES IN THIS NUMBER.
Whole Number,
CCCXXXII.
New Series.
aaanxlx. 1889.
VOL. XXVIII.
No. 8\/
Buffalo
Medical « Surgical
Journal.
Established 1845 by AUSTIN FLINT, M. D.
0
W
I
(0
J
m
D
fl.
2
0
z
I
r
<

THOS. LOTHROP, M. D.
W. W. POTTER, M. D.
(Oifors :
FLOYD S. CREGO, M. D. EDWARD B. ANGELL, M. D., Rochester, N.Y.
BUFFALO:
1889.
Entered at the Post-Office at Buffalo, N. Y., as Second-class Mail Matter.
Times Printing House, Buffalo, N. K.
				

